# Chromosome‐Level Genome Assembly for the Chinese Serow (*Capricornis milneedwardsii*) Provides Insights Into Its Taxonomic Status and Evolution

**DOI:** 10.1002/ece3.70400

**Published:** 2024-10-09

**Authors:** Anning Li, Qimeng Yang, Rongrong Li, Keli Cai, Li Zhu, Xiaoyu Wang, Gong Cheng, Xihong Wang, Yinghu Lei, Yu Jiang, Linsen Zan

**Affiliations:** ^1^ College of Animal Science and Technology Northwest A&F University Yangling Shaanxi P. R. China; ^2^ Center for Ruminant Genetic and Evolution Northwest A&F University Yangling Shaanxi P. R. China; ^3^ Research Center for the Qinling Giant Panda (Shaanxi Rare Wildlife Rescue Base) Shaanxi Academy of Forestry Sciences Zhouzhi Shaanxi P. R. China

**Keywords:** Chinese serow, chromosomal evolution, Hi‐C, HiFi, hypoxia tolerance

## Abstract

Chinese serow (*Capricornis milneedwardsii*) is mainly distributed in the south of Yellow River in China, which has been listed as vulnerable by the International Union for Conservation of Nature (IUCN). However, the reference genome of serow has not been reported and its taxonomic status is still unclear. Here, we first constructed a high‐quality chromosome‐level reference genome of *C. milneedwardsii* using PacBio long HiFi reads combined with Hi‐C technology. The assembled genome was ~2.83 Gb in size, with a contig N50 of 100.96 Mb and scaffold N50 of 112.75 Mb, which were anchored onto 24 chromosomes. Furthermore, we found that the Chinese serow was more closely related to muskox, which diverged from ~4.85 million years ago (Mya). Compared to the karyotype of goat (2*n* = 60), we found the Chinese serow (2*n* = 48) experienced six chromosome fusions, which resulted in the formation of six central centromere chromosomes. We also identified two positively selected genes (MYH6 and DCSTAMP) specific to Chinese serow, which were involved in ‘viral myocarditis’ and ‘Cardiac muscle contraction’. Interestingly, compared to other Caprinae animals, the MYH6 protein of Chinese serow occurred two mutations (E1520S and G1521S), which might be related to hypoxia tolerance. The high‐quality reference genome of *C. milneedwardsii* provides a valuable information for protection of serows and insights into its evolution.

## Graphical abstract

We constructed a high‐quality chromosome‐level reference genome of *C. milneedwardsii* for the first time. The Chinese serow was more closely related to muskox, and the karyotype of serow (2*n* = 48) occurred six chromosome fusions. Interestingly, compared to other Caprinae species, the MYH6 protein of Chinese serow occurred two mutations (E1520S and G1521S) which were conserved in Cetaceans.

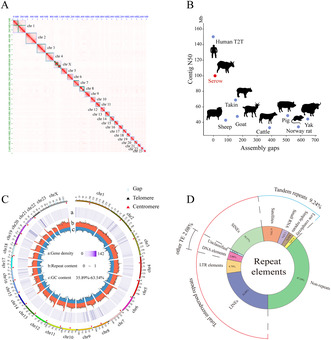

## 1 Introduction

**FIGURE 1:**
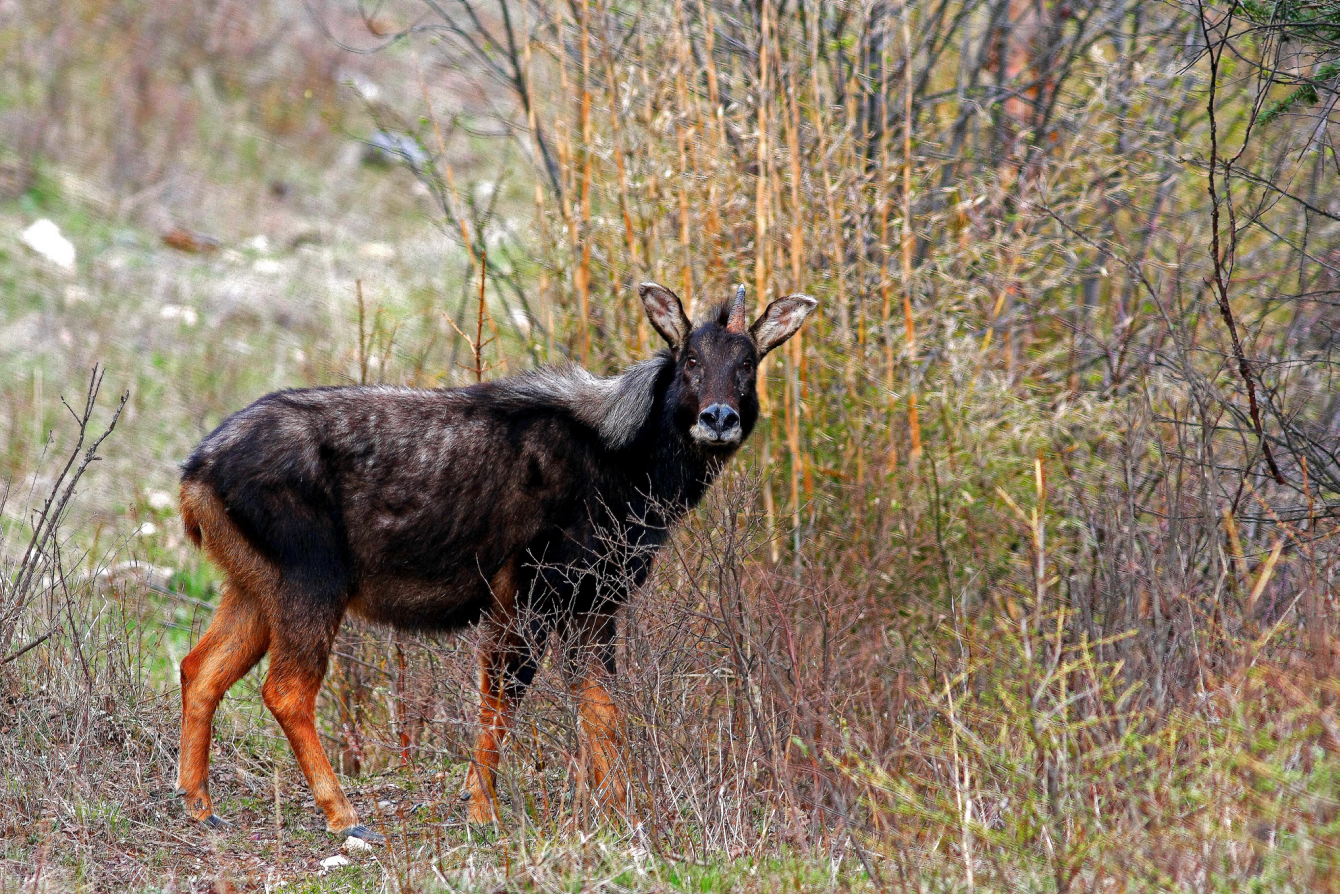
Chinese serow in Qinling Mountains (Photographer: Naxun Zhao).

The serow (*Capricornis* spp.) is a typical bovid herbivore in the tropical and subtropical regions of southeast Asia, belonging to the Caprinae subfamily (Meng et al. 2009), which has been listed as vulnerable by the IUCN (Chen et al. 2021). The *Capricornis* genus is comprised of six species: Chinese serow (*C. milneedwardsii*), Sumatran serow (*C. sumatraensis*), Formosan serow (*C. swinhoei*), Himalayan serow (*C. thar*), Japanese serow (*C. crispus*) and Red serow (*C. rubidus*) according to the Mammal Species of the World (3rd edition) (Wilson and Reeder 2005). Chinese serow, also known as Tenma, is mainly distributed in the south of Yellow River in China (Figure 1), which can be divided into five subspecies according to the physiological characteristics and geographical location (Wang 1990).

Based on the morphology, characteristic of chromosomes or analysis of mitochondrial cytochrome b genes (*Cytb*) sequences, serow (2*n* = 48) most closely related to goral (2*n* = 50) (Jantarat et al. 2017). Compared to other Caprinae animals, serow was more closely related to goral based on the mitochondrial genome sequence (Dou, Zhang, and Feng 2016; Song et al. 2023). However, the mitochondrial phylogeny might be mixed with different species (Song et al. 2023). Therefore, the phylogenetic analysis of the whole‐genomic level was required to classify the taxonomic status of serow.

In this study, we constructed a chromosome‐level reference genome of *C. milneedwardsii* using PacBio long HiFi reads combined with Hi‐C technology. Furthermore, the taxonomic status and chromosomal evolution of serow were analyzed. What's more, positive selection gene and rapid evolution gene were identified. These findings will be helpful to understand the evolution of Caprinae species.

## 2 Materials and Methods

### 2.1 Sample Collection and Ethics Statement

The liver sample from a dead adult female Chinese serow collected in December 2022 at Louguantai, Zhouzhi, Xi'an City, Shaanxi Province, China was used for genome sequencing. Sample collection and the experimental procedures were carried out according to the guidelines of the China Council on Animal Care and approved by the Experimental Animal Management Committee (EAMC) of Northwest A&F University (Approval ID: XN2023‐0619).

### 2.2 Genome Sequencing and Assembly

Obtaining HiFi reads using the PacBio Sequel II platform. First, the software HiFiAdapterFilt (v2.0.0) (Sim et al. 2022) was used with default parameters 44 bp and 97% match to remove the remaining adapter sequences from PacBio HiFi reads. Only 7 reads contaminated with adapters were removed. Second, the remaining HiFi reads were assembled *de novo* to the contig level using hifiasm (v0.19.5) with default parameters (Cheng et al. 2021). The clean Hi‐C reads were aligned to the contigs using Chromap (v0.2.4) with the additional parameter – remove‐pcr‐duplicates (Zhang et al. 2021). Third, the alignments sorted by name were used as input for YaHs (v1.2a.1) to perform genome scaffolding (Zhou, Mccarthy, and Durbin 2023). Finally, visualization and manual inspection of the Hi‐C interaction matrix were conducted in Juicebox (Durand et al. 2016). The TGS‐GapCloser (v1.2.1) (Xu et al. 2020), Pilon (v1.23) (Walker et al. 2014), BUSCO (v5.4.7) (Simao et al. 2015) and the mammalia_odb10 database were used to assess genome completeness. The distribution of GC content in the genome was determined using bedtools (v2.31.0) software with a window size of 1 Mb (Quinlan 2014). The genome resequencing was conducted using the Illumina NovaSeq6000 platform. The software GCE (v1.0.2) (https://arxiv.org/abs/1308.2012v2) was used to estimate the genome size and heterozygosity. The estimation was based on 40× Illumina WGS reads using the k‐mer method with a k‐mer size of 17.

### 2.3 Repeats and Gene Annotation

RepeatMasker (v4.1.5) (https://www.repeatmasker.org) and repeat database (including Dfam v.3.7 and RepBase library October 26, 2018) were used to identify repeats in the serow genome. The software Liftoff (v1.6.3) (Shumate and Salzberg 2021) was utilized to map the annotation of the sheep genome (ARS‐UI_Ramb_v2.0_GCF_016772045.1, annotation‐source NCBI Ovis aries Annotation Release 104) onto the serow genome. The parameters were polish, copies, and flank 0.2. The input comprised the genome sequences of sheep and serow, along with the GFF‐formatted annotation of the sheep genome. Minimap2 (v2.26‐r1175) (Li 2018) was used to align the entire gene sequence, including exons and introns, to the target, resulting in the gene annotation file of the serow, from which protein‐coding genes were filtered.

The Circos (v0.69–9) software (Krzywinski et al. 2009) was used to visualize the details of the serow genome. The outermost track represents chromosome lengths, followed by gene density, repetitive sequences, and GC content, each with a window size of 1 Mb. Centromere: 50,000 bp was defined as the longest merging distance between the two sections, and the location of centromere on each chromosome was found with the interval span greater than 200,000 bp as the filter condition. Telomeres: Telomere positions on each chromosome were determined using a filtering criterion of telomeric repeat sequences extending at least 500 bp from the chromosome ends. Additionally, we assessed the presence of telomeric repeat sequence (TTAGGG) within the terminal 20 kb of the autosomal telomeric sequences.

Using the AGAT (v1.2.0) software to process annotation results, the agat_sp_keep_longest_isoform.pl. script was executed to extract the annotation file of the longest transcript sequences from the annotation file of the serow genome. The agat_sp_extract_sequences.pl. script was executed to obtain the protein sequences of the longest transcripts. CDSs (average CDSs length per gene) and exon average length were calculated from the longest transcription sequence annotation file of serow. According to the longest transcription sequence annotation file of serow, the intron sequence position is obtained by taking the difference set between gene sequence position and exon sequence, and then its average length is calculated.

### 2.4 Mitochondrial Genome Assembly

The MitoHiFi (v2.2) software (Uliano‐Silva et al. 2023) was used to assemble the mitochondrial genome from contigs using the serow mitochondrial genome NC_023457.1 as a reference. Mitochondrial sequences were retrieved from contigs using BLASTn, and sequences with alignment length > 80% of mitochondrial length and identical matches > 90% were identified as mitochondrial sequences. All 64 sequences identified were small contig (< 60 kb) that did not interact with other sequences. The mitochondrial genome was annotated with MitoFinder (v1.4) (Allio et al. 2020) and visualized by OGDRAW (Greiner, Lehwark, and Bock 2019).

### 2.5 Phylogenetic Analysis and Divergence Time Estimation

The phylogenetic tree was constructed including 18 mitochondrial genomes: *C. milneedwardsii* (OR_551470), *Ovis aries* (NC_001941.1), *Ovis ammon* (KT781689.1), *Ovis nivicola* (NC_039431.1), *Capra sibirica* (NC_020626.1), *Capra hircus* (MZ073671.1), *Capra aegagrus* (NC_028161.1), *Oreamnos americanus* (NC_020630.1), *Budorcas taxicolor* (NC_013069.1), *Ovibos moschatus* (NC_020631.1), *Pantholops hodgsonii* (NC_007441.1), *Bos taurus* (NC_006853.1), *Hemitragus hylocrius* (the mitochondrial genome was assembled based on the Illumina WGS sequencing data SRR11430235), *Pseudois nayaur* (NC_020632.1), *Ammotragus lervia* (NC_009510.1), *Ovis orientalis* (NC_026063.1), *Naemorhedus goral* (NC_021381.1) and *Rupicapra rupicapra* (NC_020633.1). Mitochondrial genome was applied to MEGA (v11) (Tamura, Stecher, and Kumar 2021). The ClustalW was used for sequence alignment, and the Maximum Likelihood method was selected with the bootstrap value set to 100 to construct the mitochondrial phylogenetic tree.

The genomes of *Pantholops hodgsonii* (GCF_000400835.1), *Oreamnos americanus* (GCA_009758055.1), *Ovibos moschatus* (GCA_021462335.1), *Ovis ammon* (GCA_028583565.1), *Capra aegagrus* (GCA_000978405.1), *Capra hircus* (GCA_015443085.1), *C. milneedwardsii* (serow.2 genome, GCA_032405125.2), *Ovis aries* (GCF_016772045.1), *Capra sibirica* (GCA_003182615.2), *Ovis nivicola* (GCA_903231385.1), *Budorcas taxicolor* (GCF_023091745.1), *Hemitragus hylocrius* (GCA_004026825.1), *Pseudois nayaur* (GCA_003182575.1), *Ammotragus lervia* (GCA_002201775.1), *Ovis orientalis* (GCA_014523465.1), and *Rupicapra rupicapra* (GCA_963981305.1) were separately aligned to the genome of *Bos Taurus* (GCF_002263795.1) using the LAST (v1409) (Kielbasa et al. 2011). The comparison results were then combined with Multiz (v11.2) (Blanchette et al. 2004) and filtered with Gblocks (v0.91b), retaining 4.31 million quadruple degenerate sites. A species tree was then constructed using RAxML‐NG (v1.2.0) (Kozlov et al. 2019) under the GTR + G model, with bootstrap replicates set to 100. The BASEML subroutine in the PAML (v4.10.7) package (Yang 2007) was used to calculate the neutral replacement rate (0.197217 + −0.000417). The calculated neutral replacement rate was used to calculate the divergence time for MCMCTREE, and the time calibration was performed on both nodes using the previously studied calibration time. The divergence between cattle and other animals is ‘> 18.3 < 28.5’ Mya, the divergence between goats and sheep is ‘> 3.9 < 8.1’ Mya (Yang 2007).

### 2.6 Chromosomal Evolution

The LAST (version 1409) (Kielbasa et al. 2011) was used to align the goat genome (GCA_015443085.1) to the takin (GCF_023091745.1) and the assembled serow genome to the goat genome, respectively. Using MCScan (v1.3.6) (Tang et al. 2024), a fragment length is more than 30,000 bp for filter, linear relation between visualization on two groups.

### 2.7 Heterozygosity Estimation

The genome sequencing data was compared with the assembled genome using BWA‐MEM (v0.7.17). The SAMtools (v1.17) software (Li et al. 2009) was used to convert SAM files to BAM files. GATK (v4.1.8.1) software (Mckenna et al. 2010) was used to invoke and screen single nucleotide polymorphisms (SNPs). SNPs with mutation type 0/1 were screened out, and then the number of SNPs was compared with the length of the reference genome to obtain the heterozygosity of the genome. Heterozygosity of other species was obtained from previous research (Liu et al. 2021).

### 2.8 Positive Selection Gene, Rapid Evolution Gene Identification

The CODEML subroutine in PAML was used to identify positive selection genes (PSGs) and rapid evolution genes (REGs) in the serow genome. Serow branches in phylogenetic trees were designated as foreground branches for independent positive selection analysis. The PSG was determined by the branch‐site model, where the alternative model allowed a positive selection of the site on the foreground branch. REG was tested with the branch model, where the alternative model allowed for different rates for different branches. Models allowing positive selection (dN/dS > 1) and empty models allowing neutral evolution and purification selection (dN/dS < 1) were compared using the likelihood ratio test (LRT). KOBAS (v3.0) software (Bu et al. 2021) and DAVID software were used for KEGG and GO pathway enrichment analysis of the identified positive selection genes and rapid evolution genes (*p* < 0.05).

### 2.9 Prediction of the Three‐Dimensional Structure of MYH6 Protein

Download the amino acid sequence of the bovine *MYH6* gene from NCBI, the software SMART (Simple Modular Architecture Research Tool) (https://smart.embl‐heidelberg.de/) was used to predict the functional domain in the protein sequence. The nucleotide sequences of *MYH6* gene were converted into amino acid sequences using the CODEML subroutine in PAML (v4.10.7) (Yang 2007). The converted amino acid sequences were subjected to multiple sequence alignment using the Clustal Omega (version 1.2.4) tool available in the EMBOSS software (https://www.ebi.ac.uk/Tools/emboss/). Download the amino acid sequence of the human *MYH6* gene from NCBI. Visualize the retrieved three‐dimensional structure from the database using PyMOL (version2.5.7) (https://pymol.org/). Mutate the amino acids at positions 1520 and 1521 from E and G to S and S, respectively. Visualize the hydrogen bonds formed between the surrounding atoms and the two amino acids before and after the mutation. Utilize the software ProtScale (https://web.expasy.org/protscale/) to analyze the hydrophobicity of the two positions before and after the mutation and their adjacent amino acids.

## 3 Results

### 3.1 Chromosome‐Level de novo Genome Assembly of Chinese serow

To estimate the genome size of *C. milneedwardsii*, 120.10 Gb clean reads were used for k‐mer analysis. The genome size was estimated at 2.88 Gb with 0.43% heterozygosity (*k* = 17, Figure S1). A total of 107.2 Gb HiFi reads (~37×) were generated by the PacBio Sequel II platform (Table S1). We assembled a ~2.83 Gb reference genome with 240 contigs and a contig N50 of 100.96 Mb using Hifiasm v0.19.5 (Table 1).

**TABLE 1:** Assembly statistics of Chinese serow genome.

Assembly	Number/length
Total assembly length (bp)	2,828,266,837
Gap number	6
Number of contigs	165
N50 contig length (bp)	100,955,055
Contig L50	11
Number of scaffolds	158
N50 scaffold length (bp)	112,748,084
Scaffold L50	9
Chromosome number	23 + X
GC content (%)	43.07
Protein‐coding genes	20,783

**FIGURE 2:**
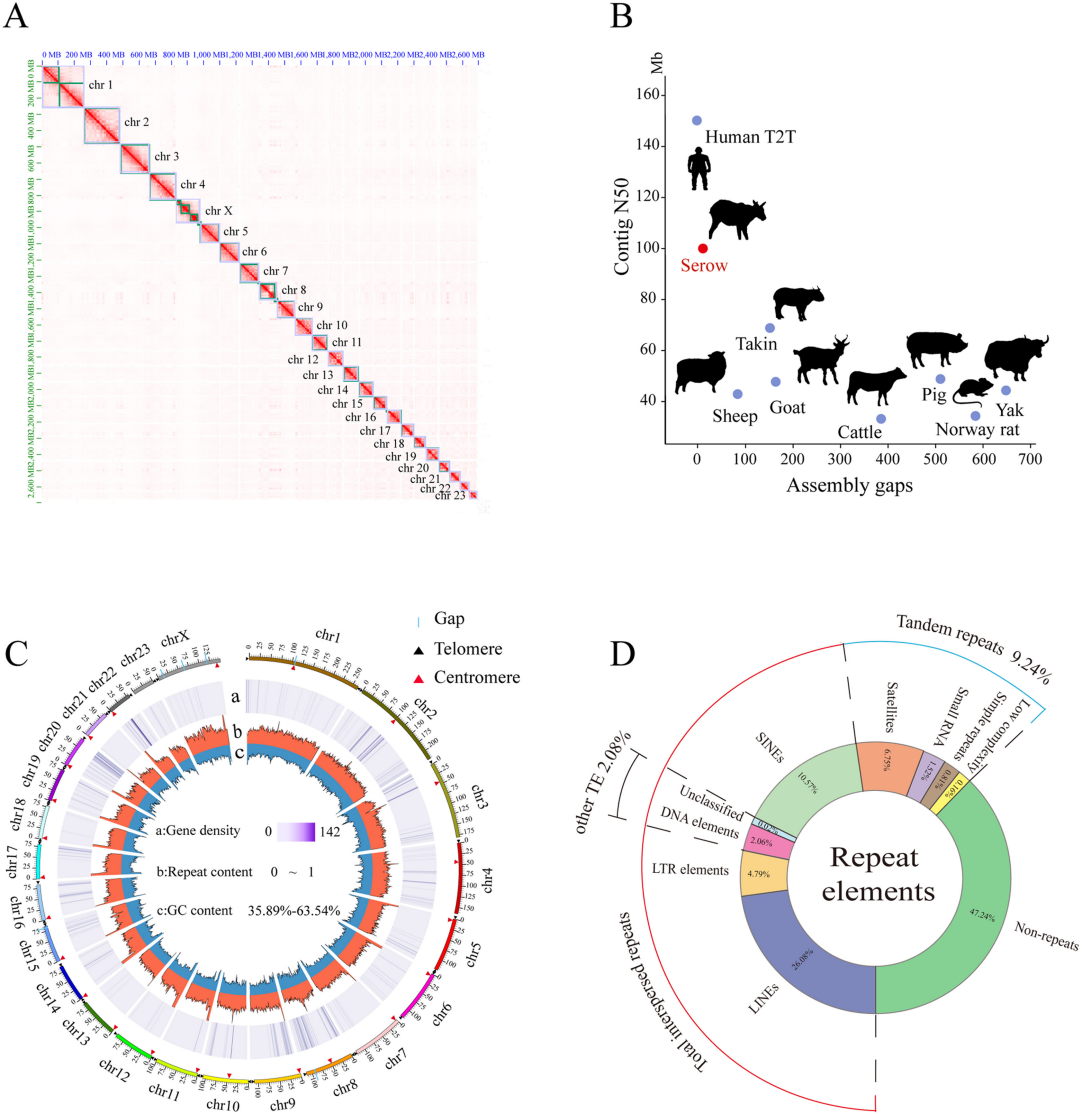
Overview of *C. milneedwardsii* genome assembly. (A) The Hi‐C interaction heatmap of Chinese serow. (B) Circos plot showing the visualization of genomic details with a window of 1 Mb. a. gene density; b. repeat content; c. GC content. (C) The contig N50 and the number of gaps of different domestic and wild animals. (D) The ratio of repeat sequences in Chinese serow genome.

To obtain a chromosome‐level assembly, 352.60 Gb Hi‐C reads (~120×) spanned to 252 scaffolds with a N50 of 112.75 Mb (Table S2), which anchored onto 24 chromosomes (Figure 2A). The gap in Chr 7 was filled by TGS‐GapCloser v1.2.1. After deleting the mitochondrial genome sequences and polishing with pilon v1.23, the completeness of final assembled genome reached 95.90% with six gaps using cetartiodactyla_odb10 database in BUSCO v5.4.7 (Figure 2B, Table S3). Compared to the references of other species, Chinese serow had a higher contig N50 and fewer gaps, with the exception of humans (Figure 2C), which indicated we assembled a high‐quality chromosome‐level reference genome of Chinese serow.

The size of assembled mitochondrial genome was about 16,461 bp (Figure S2) (OR551470), which shared 99.02%, 99.00%, 98.60% and 97.74% sequence similarity with Himalayan serow (KT345703.1), Sumatran serow (NC_020629.1), Chinese serow (NC_023457.1) and Red serow (NC_045205.1), respectively.

### 3.2 Genome Annotation

Repeat sequences accounted for 51.28% of the assembled genome, dominated by long interspersed nuclear elements (LINEs, 26.08%), followed by short interspersed nuclear elements (SINEs, 10.57%), satellites (6.75%), long terminal repeats (LTRs, 4.79%), and DNA elements (2.06%) (Figure 2D, Table S4). In addition, 518,543 simple repeats (0.81%) were identified (Table S4). The dimers were the largest proportion (31.14%) of SSRs, followed by quadmers (15.81%), monomers (14.08%), pentamers (10.57%), hexamers (10.04%) and trimers (9.58%) (Table S5). 20,783 protein‐coding genes were annotated by the homology‐based method. The average gene length was 40,325 bp with 8.20 exons on average.

Furthermore, 11,949,425 SNPs were identified by GATK software (v4.1.8.0), which was used to calculate the heterozygosity. The genome heterozygosity of Chinese serow was about 0.00422, which was lower than Steenbok, Springbuck and African Buffalo, while it was higher than that of Nilgiri Tahr, Mountain Nyala and Bighorn sheep (Figure S3) (Liu et al. 2021).

### 3.3 Phylogenetic Analysis at the Mitochondrial and Genomic Level

To classify the taxonomic status of Chinese serow, we constructed the phylogenetic tree at the mitochondrial and genomic level, respectively. Chinese serow was closely related to goral at the mitochondrial genomic level (Figure S4), which was consistent with previous study (Song et al. 2023).

**FIGURE 3:**
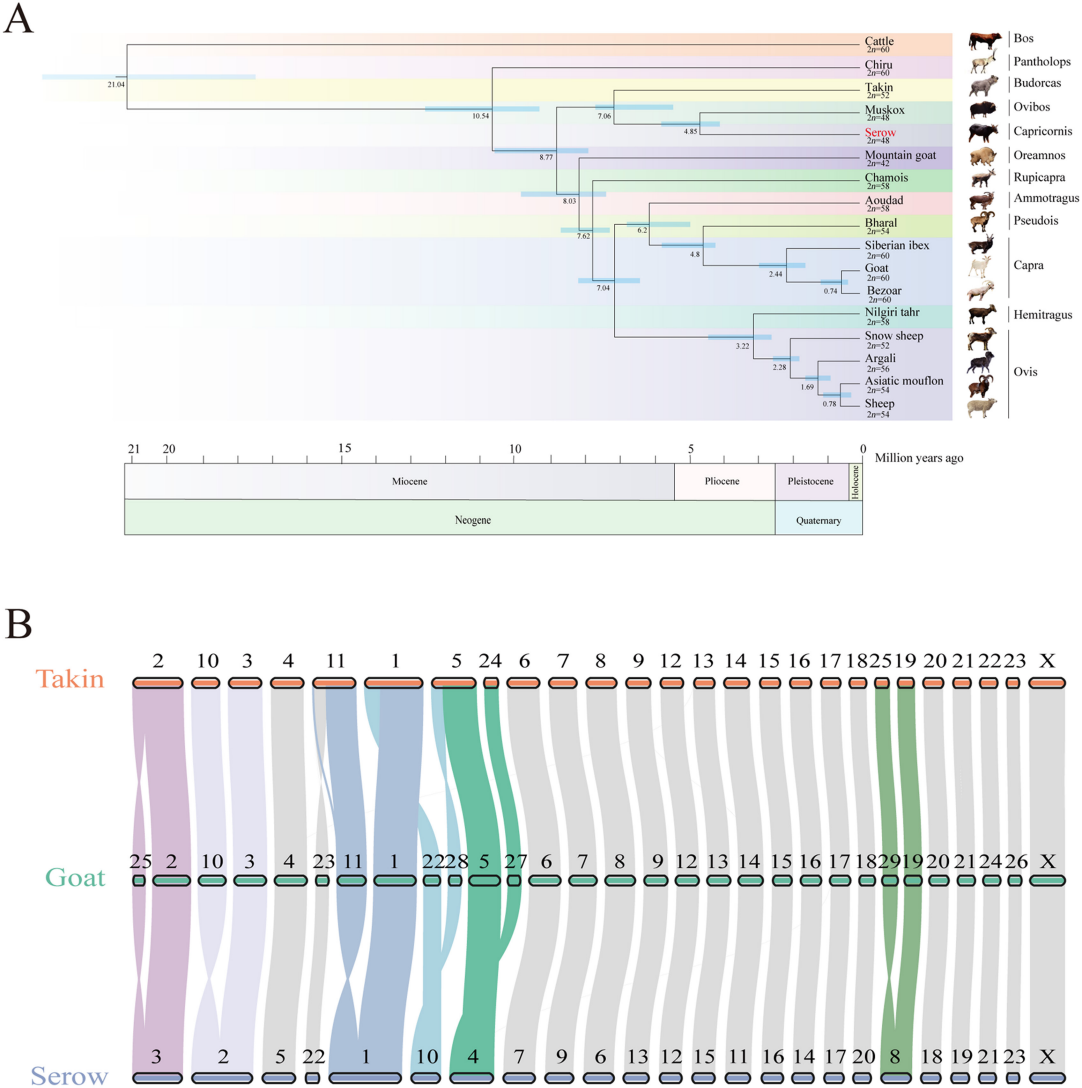
Phylogenetic and chromosomal evolution analysis. (A). Phylogenetic analysis at the genomic level. (B) The collinear relationship of Takin, Goat and Chinese serow.

At the genomic level, the caprinae subfamily was divided into two branches at ~8.77 Mya. One branch consisted with Chinese serow, muskox and takin. Chinese serow was closely related to muskox rather than takin. Chinese serow had split from the ancestor of takin at ~7.06 Mya, while diverged from the ancestor of muskox at ~4.85 Mya (Figure 3A). The other subfamily caprinae species made up another branch. What's more, the ancestor of goat and sheep was more closely related to chamois, rather than mountain goat in our previous study (Li, Yang et al. 2023).

### 3.4 Chromosome Evolution Analysis

Compared to the karyotype of goat (2*n* = 60), the karyotype of Chinese serow (2*n* = 48) occurred six fusions. These fusions were from Chr25 and 2 to Chr3, Chr10 and 3 to Chr2, Chr11 and 1 to Chr1, Chr22 and 28 to Chr10, Chr5 and 27 to Chr4, Chr29 and 19 to Chr8, respectively (Figure 3B). Compared to the karyotype of goat (2*n* = 60), the karyotype of takin (2*n* = 52) occurred four fusions, which was consistent with our previous study (Li, Yang et al. 2023).

### 3.5 Identification and Analysis of REGs and PSGs

**FIGURE 4:**
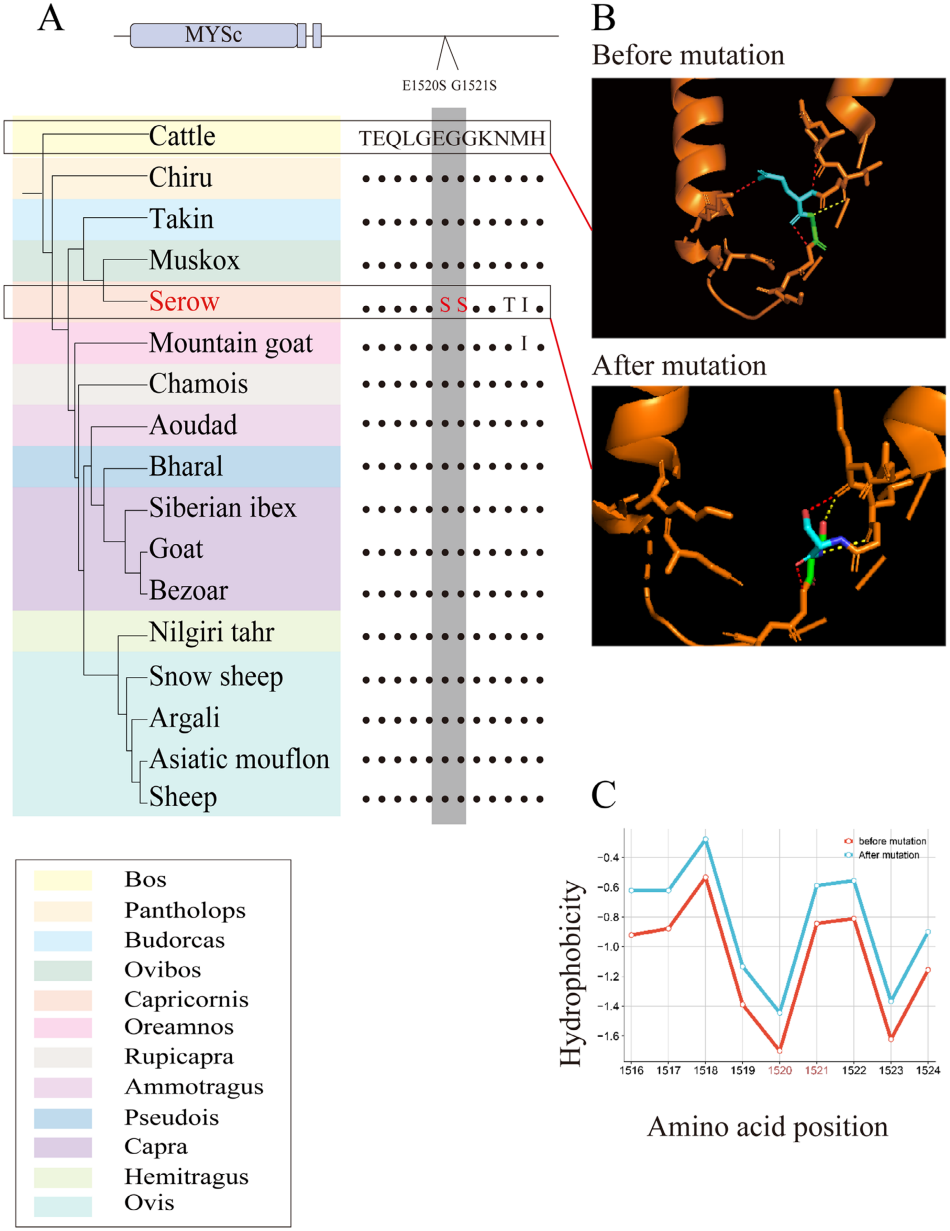
The two mutations of MYH6 protein in Chinese serow. (A) The two mutations of E1520S and G1521S in Chinese serow was different from other Caprinae animals; (B) Changes in 3D structure before and after mutations; (C) Changes in hydrophobicity before and after mutations. Light blue color: 1520aa; Light green color: 1521aa; Red color: The hydrogen bond between the two α‐helices; Yellow color: The hydrogen bond.

To identify the rapidly evolving genes (REGs) and positively selected genes (PSGs), the Codeml program in PAML (v4.9j) with the branch model and branch‐site model was performed. A total of 1119 REGs were identified in Chinese serow (Table S6), which were functionally enriched in ‘Metabolic pathways’ and ‘Fatty acid metabolism’ (Figure S5A). In addition, two PSGs (MYH6 and DCSTAMP) were identified in Chinese serow, which were involved in ‘viral myocarditis’ and ‘Cardiac muscle contraction’ (Figure S5B). Interestingly, there are two mutations (E1520S and G1521S) presented in the MYH6 protein of Chinese serow, which were not existed in other Caprinae animals (Figure 4A). The mutations of E1520S and G1521S caused the hydrogen bond between the two α‐helices to disappear (Figure 4B) and reduced the hydrophobicity of amino acids (Figure 4C) (Li, Li et al. 2023), which might change the stability of the MYH6 protein. Furthermore, the homology alignment of the MYH6 protein showed that the mutations of E1520S and G1521S were conserved between Chinese serow and Cetaceans (Common Bottlenose Dolphin, killer whale, Beluga whale, Sperm whale, Blue whale and Minke whale) (Figure S6) (Fu et al. 2022), which indicated the two mutations might be related to hypoxia tolerance.

## 4 Discussion

### 4.1 Taxonomic Status of Serow

In this study, the serow was most closely related to goral at the mitochondrial genomic level, which was consistent with previous studies (Dou, Zhang, and Feng 2016; Jantarat et al. 2017; Song et al. 2023). However, at the genomic level, the serow was most closely related to muskox in our findings. Recently, it was reported that the goral was more closely related to takin compared to muskox (Sun et al. 2024). Because of the lacking of the goral reference genome in the public databases, the goral was not presented in the phylogenetic tree. It has been reported that the muskox was diverged from *Hezhengia bohlini* which were found in the Hezheng region of Gansu Province, China (Qiu, Wang, and Xie 2000). Based on the characters of skull, teeth and horn core, serow was closely related to muskox and takin (Shi and Deng 2021). So we speculated that serow was more closely related to muskox, goral and takin than other Caprinae species which were originated in East Asia. Then, muskox migrated to the North Pole. Furthermore, it was divided into two branches. One branch was serow and muskox, whereas another was takin and goral.

### 4.2 Chromosome Evolution of Serow

Chromosome evolution has been reported to link with phenotypic evolution and new speciation (Damas, Corbo, and Lewin 2021; Eichler and Sankoff 2003). Because of the lacking of the chromosome‐level reference genome of goral and muskox, we only compared the karyotype of goat (2*n* = 60), takin (2*n* = 52) and serow (2*n* = 48). Compared to the karyotype of goat, the karyotype of serow occurred six fusions resulting in a decrease in the number of serow chromosomes to 2*n* = 48. We speculated that the chromosome fusion resulted in the formation of central centromere chromosome, which was consistent with the karyotype of serow including six central centromere chromosomes and 18 terminal centromere chromosomes (Soma et al. 1982). In addition, the formation of new species was speeded up by chromosome fusions (Yin et al. 2021). Recently, it was reported that a sustainable mouse karyotype created by chromosome fusion (Wang et al. 2022), the karyotype of which was reduced from 2*n* = 40 to 2*n* = 38. Therefore, further research on chromosome fusions is helpful to explore the chromosome evolution.

### 4.3 Adaptive Evolution of Serow

The Chinese serow inhabited alpine forest area at altitudes from 1400 to 3400 m, which was similar with takin (Li et al. 2021). A large number of REGs identified were mainly enriched in metabolism pathways and two PEGs identified were involved in cardiac muscle contraction, which were consistent with the strong ability of climb cliffs in Chinese serow (Li et al. 2021). Interestingly, two mutations (E1520S and G1521S) of MYH6 protein in Chinese serow were not existed in other Caprinae animals. However, the mutations of E1520S and G1521S were conserved in Cetaceans. Because Chinese serow and Cetaceans have a strong tolerance to hypoxia, we speculated the mutations of E1520S and G1521S in MYH6 protein related to hypoxia tolerance and myocarditis. It is required to further research because there was no enough evidence to support this hypothesis.

## 5 Conclusion

We assembled a high‐quality chromosome‐level reference genome of *C. milneedwardsii*. Chinese serow was most closely related to muskox, diverged from ~4.85 Mya. Compared to the karyotype of goat (2*n* = 60), the Chinese serow (2*n* = 48) experienced six chromosome fusions. Interestingly, we identified a positively selected gene, MYH6 (two mutation sites: E1520S and G1521S), conserved in the Cetaceans, but not in the other Caprinae animals. These results provide a valuable information for protection of serows and insights into its evolution.

## Author Contributions

**Anning Li:** formal analysis (equal), funding acquisition (equal), project administration (equal), supervision (equal), writing – original draft (equal). **Qimeng Yang:** formal analysis (equal), writing – review and editing (supporting). **Rongrong Li:** formal analysis (equal), writing – review and editing (supporting). **Keli Cai:** data curation (equal). **Li Zhu:** data curation (equal). **Xiaoyu Wang:** resources (equal). **Gong Cheng:** resources (equal). **Xihong Wang:** resources (equal), writing – review and editing (equal). **Yinghu Lei:** resources (equal). **Yu Jiang:** project administration (equal), supervision (equal), writing – review and editing (equal). **Linsen Zan:** funding acquisition (equal), resources (equal), writing – review and editing (equal).

## Conflicts of Interest

The authors declare no conflicts of interest.

## Supporting information


**Figure S1.** K‐mer analysis of the genome size by gce v1.0.2.
**Figure S2.** The mitochondrial genome of Chinese serow.
**Figure S3.** The genome heterozygosity of different domestic and wild animals.
**Figure S4.** Phylogenetic analysis at the mitochondrial genomic level.
**Figure S5.** KEGG enrichment analysis.
**Figure S6.** The homology alignment of the MYH6 protein.
**Table S1.** Summary of HiFi reads.
**Table S2.** Summary of Hi‐C reads.
**Table S3.** Assessment of genome completeness.
**Table S4.** Repeat content of Chinese serow.
**Table S5.** Simple repeats content of Chinese serow.

**Table S6.** The list of the rapidly evolving genes.

## Data Availability

The data presented in this study are available in the Sequence Read Archive (SRA) database, BioProject: PRJNA1002856. The serow genome has been submitted to the GenBank database (GenBank accession No. JAXIOT000000000). Mitochondrial genome has been submitted to the GenBank database (GenBank accession OR551470).
