# Supplementary material for: Chromosome‐Level Genome Assembly for the Chinese Serow (Capricornis milneedwardsii) Provides Insights Into Its Taxonomic Status and Evolution
Source: Ecol Evol. 2024 Oct 9;14(10):e70400. doi: 10.1002/ece3.70400 (PMC11462074; doi:10.1002/ece3.70400)
Supplement: Supplementary file 1 — Figure S1. K‐mer analysis of the genome size by gce v1.0.2. Figure S2. The mitochondrial genome of Chinese serow. Figure S3. The genome heterozygosity of different domestic and wild animals. Figure S4. Phylogenetic analysis at the mitochondrial genomic level. Figure S5. KEGG enrichment analysis. Figure S6. The homology alignment of the MYH6 protein. Table S1. Summary of HiFi reads. Table S2. Summary of Hi‐C reads. Table S3. Assessment of genome completeness. Table S4. Repeat content of Chinese serow. Table S5. Simple repeats content of Chinese serow. [file ECE3-14-e70400-s002.docx]

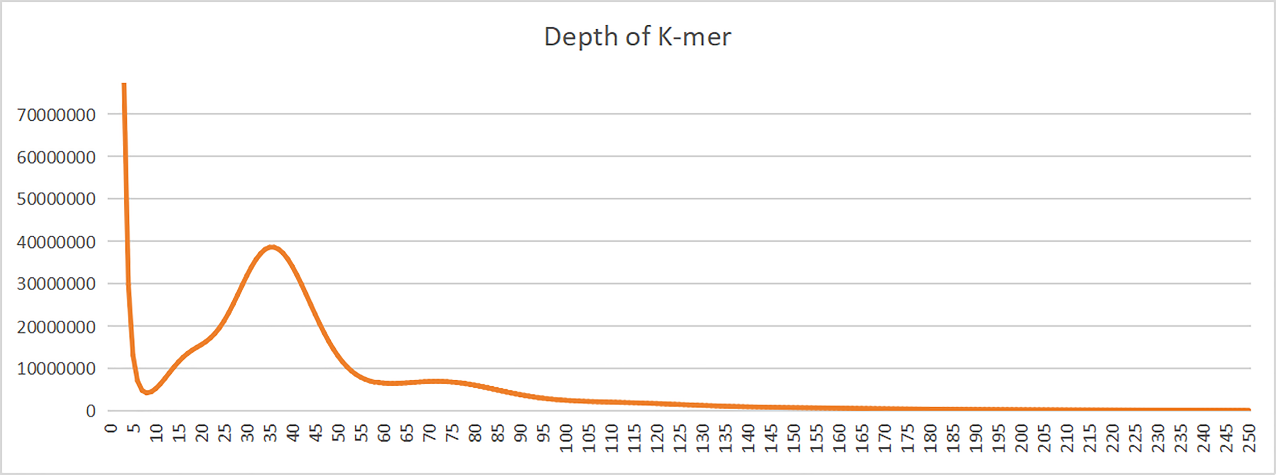


**Figure S1.** K-mer analysis of the genome size by gce v1.0.2 (<https://arxiv.org/abs/1308.2012v2>).


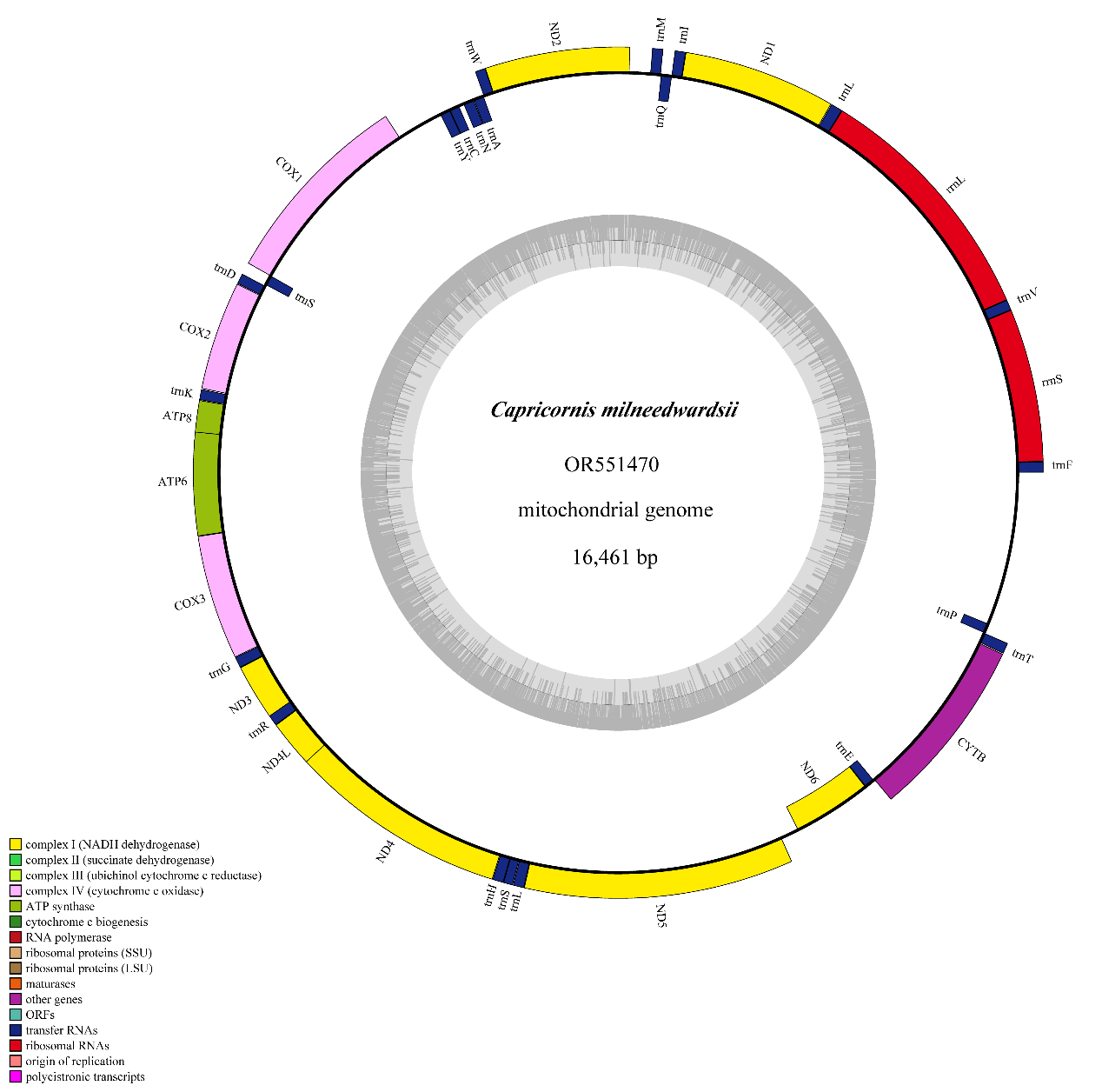


**Figure S2.** The mitochondrial genome of Chinese serow.


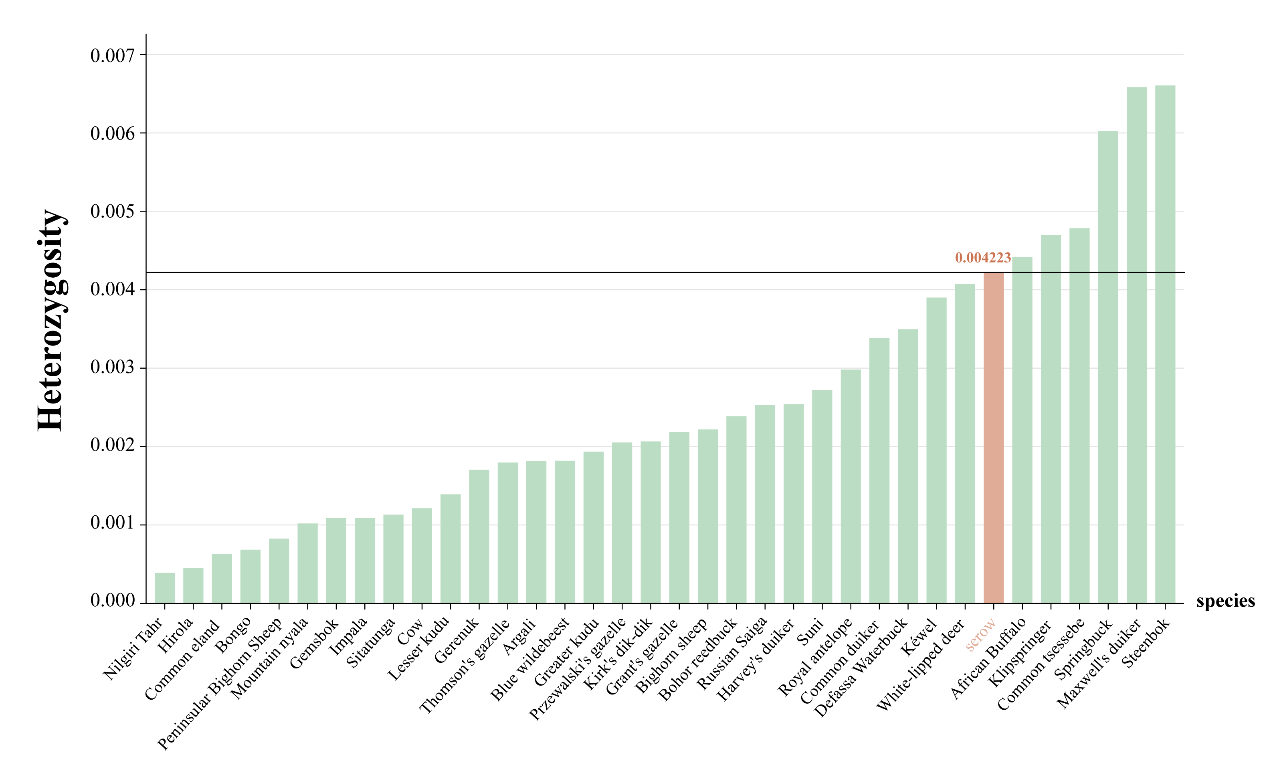


**Figure S3.** The genome heterozygosity of different domestic and wild animals. The heterozygosity rates of other species were reported in previous reference (Liu et al., 2021).

**Figure S4.** Phylogenetic analysis at the mitochondrial genomic level. The Maximum Likelihood method with 100 bootstrap replicates using MEGA v11.


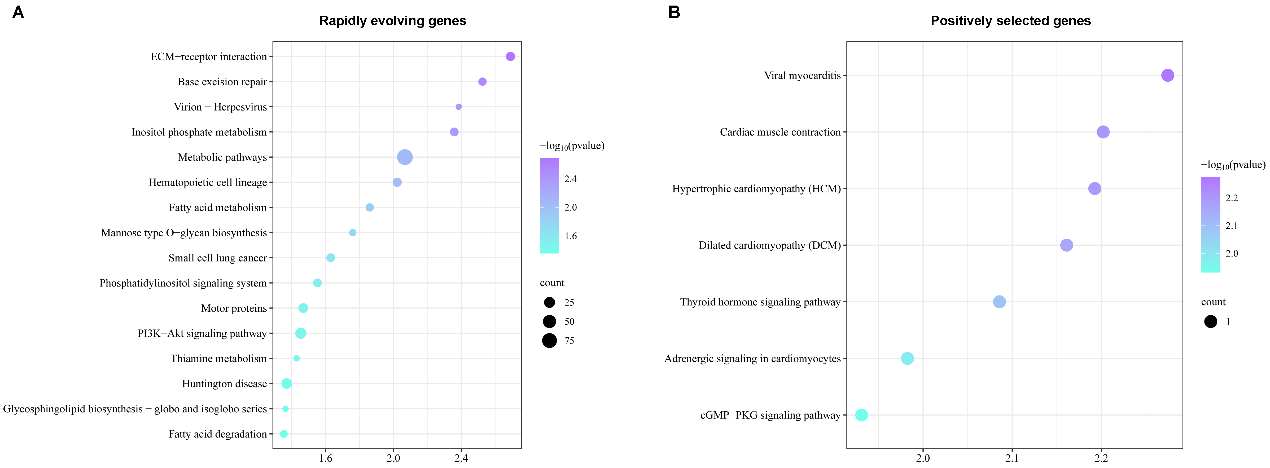


**Figure S5.** KEGG enrichment analysis.

A. Rapidly evolving genes; B. Positively selected genes.

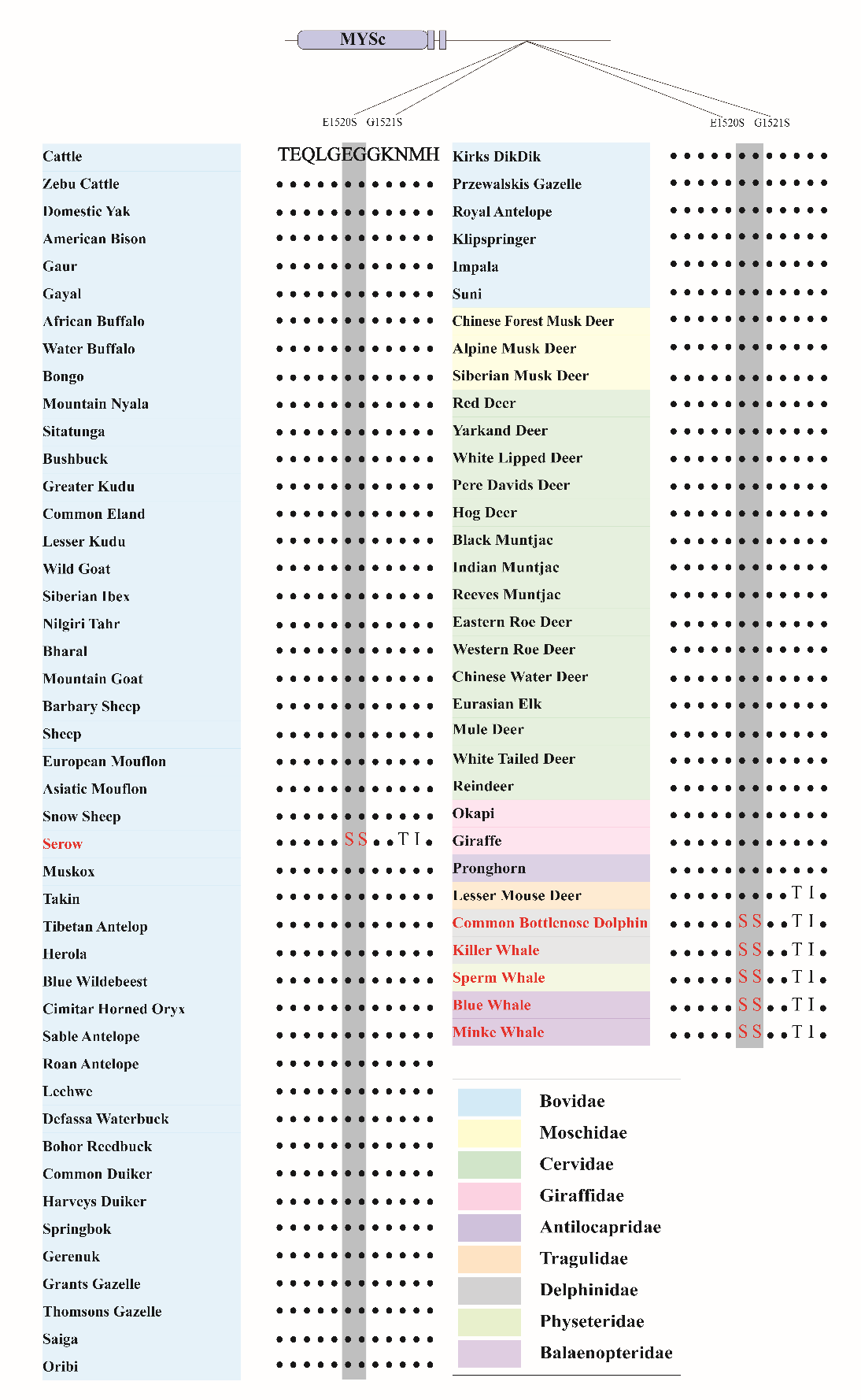


**Figure S6.** The homology alignment of the MYH6 protein (Fu et al., 2022).

MYSc: functional domain of MYH6.

**Table S1.** Summary of HiFi reads

|  | **Total reads** | **Total bases** | **Average length** |
| --- | --- | --- | --- |
| **Cell 1** | 2,427,935 | 34,549,237,725 | 14,230 |
| **Cell 2** | 2,217,929 | 35,699,072,040 | 16,096 |
| **Cell 3** | 2,290,565 | 36,986,452,117 | 16,147 |
| **Total** | **6,936,429** | **107,234,761,882** | **15,460** |

**Table S2.** Summary of Hi-C reads

|  | **Total reads** | **Total bases** | **Q20** | **Q30** |
| --- | --- | --- | --- | --- |
| **R1** | 1,176,212,303 | 176,309,117,607 | 97.12% | 91.99% |
| **R2** | 1,176,212,303 | 176,310,267,395 | 95.73% | 88.90% |

**Table S3.** Assessment of genome completeness

| **Types of BUSCOs** | **Count** | **Ratio (%)** |
| --- | --- | --- |
| Complete BUSCOs (C) | 12,799 | 95.9 |
| Complete and single-copy BUSCOs (S) | 12,541 | 94.0 |
| Complete and duplicated BUSCOs (D) | 258 | 1.9 |
| Fragmented BUSCOs (F) | 134 | 1.0 |
| Missing BUSCOs (M) | 402 | 3.1 |
| Total BUSCO groups searched | 13,335 | -- |

**Table S4.** Repeat content of Chinese serow

|  | number of elements | length occupied  (bp) | percentage of sequence (%) |
| --- | --- | --- | --- |
| SINEs | 2,045,903 | 299,209,866 | 10.57 |
| LINEs | 1,326,933 | 738,052,412 | 26.08 |
| LTR elements | 414,999 | 135,506,468 | 4.79 |
| DNA elements | 294,129 | 58,215,116 | 2.06 |
| Unclassified | 3,116 | 476,587 | 0.02 |
| Total interspersed repeats | -- | 1,231,460,449 | 43.52 |
| Small RNA | 253,448 | 43,056,713 | 1.52 |
| Satellites | 55,454 | 190,883,219 | 6.75 |
| Simple repeats | 518,543 | 22,996,196 | 0.81 |
| Low complexity | 82,308 | 4,391,263 | 0.16 |

**Table S5.** Simple repeats content of Chinese serow

| **Repeat type** | **Unit size (repeat number)** | **Number** | **Ratio**  **(%)** |
| --- | --- | --- | --- |
| mono-nucleotide | 1 (≥10) | 73,614 | 14.08 |
| Di-nucleotide | 2 (≥6) | 162,787 | 31.14 |
| Tri-nucleotide | 3 (≥5) | 50,084 | 9.58 |
| Tetra-nucleotide | 4 (≥5) | 82,633 | 15.81 |
| Penta-nucleotide | 5 (≥5) | 55,234 | 10.57 |
| Hexa-nucleotide | 6 (≥5) | 52,461 | 10.04 |

**Table S6.** The list of the rapidly evolving genes (**Shown in the supplemental file**)

References

Fu WW, Wang R, Nanaei HA, et al. 2022. RGD v2.0: a major update of the ruminant functional and evolutionary genomics database. *Nucleic Acids Research*, **50**(D1): D1091-D1099.

Liu SL, Westbury MV, Dussex N, et al. 2021. Ancient and modem genomes unravel the evolutionary history of the rhinoceros family. *Cell*, **184**(19): 4874-4885.
